# Rapid and Accurate Identification of SARS-CoV-2 Variants Using Real Time PCR Assays

**DOI:** 10.3389/fcimb.2022.894613

**Published:** 2022-05-10

**Authors:** Gwynngelle A. Borillo, Ron M. Kagan, Elizabeth M. Marlowe

**Affiliations:** Infectious Diseases R&D, Quest Diagnostics, San Juan Capistrano, CA, United States

**Keywords:** SARS-CoV-2, COVID-19, variants, genotyping, RT-PCR

## Abstract

**Background:**

Genomic surveillance efforts for SARS-CoV-2 are needed to understand the epidemiology of the COVID-19 pandemic. Viral variants may impact routine diagnostic testing, increase viral transmissibility, cause differences in disease severity, have decreased susceptibility to therapeutics, and/or confer the ability to evade host immunity. While viral whole-genome sequencing (WGS) has played a leading role in surveillance programs, many laboratories lack the expertise and resources for performing WGS. This study describes the performance of multiplexed real-time reverse transcription-PCR (RT-PCR) assays for identification of SARS-CoV-2 variants.

**Methods:**

SARS-CoV-2 specimens were tested for spike-gene variants using a combination of allele-specific primer and allele-specific detection technology (PlexPrime^®^ and PlexZyme^®^). Targeted detection of spike gene mutations by RT-PCR was compared to variant detection in positive specimens by WGS, including the recently emerged SARS-CoV-2 Omicron variant.

**Results:**

A total of 398 SAR-CoV-2 RT-PCR positive and 39 negative specimens previously tested by WGS were re-tested by RT-PCR genotyping. PCR detection of spike gene mutations N501Y, E484K, and S982A correlated 100% with WGS for the 29 lineages represented, including Alpha (B.1.1.7), Beta (B.1.351), and Gamma (P.1). Incorporating the P681R spike gene mutation also allowed screening for the SARS-CoV-2 Delta variant (B.1.617.2 and AY sublineages). Further sampling of 664 specimens that were screened by WGS between June and August 2021 and then re-tested by RT-PCR showed strong agreement for Delta variant positivity: 34.5% for WGS vs 32.9% for RT-PCR in June; 100% vs 97.8% in August. In a blinded panel of 16 Omicron and 16 Delta specimens, results of RT-PCR were 100% concordant with WGS results.

**Conclusions:**

These data demonstrate that multiplexed real-time RT-PCR genotyping has strong agreement with WGS and may provide additional SARS-CoV-2 variant screening capabilities when WGS is unavailable or cost-prohibitive. RT-PCR genotyping assays may also supplement existing sequencing efforts while providing rapid results at or near the time of diagnosis to help guide patient management.

## Introduction

Coronavirus disease 2019 (COVID-19), caused by the severe acute respiratory syndrome coronavirus 2 (SARS-CoV-2), emerged in the Chinese province of Wuhan in November 2019 (Wu et al., 2020; Zhu et al., 2020) and was declared a global pandemic by the World Health Organization (WHO) after its rapid spread around the world (https://www.who.int/emergencies/diseases/novel-coronavirus-2019). As of March 7, 2022, over 447 million COVID-19 cases have been reported worldwide, resulting in over 6 million deaths (https://coronavirus.jhu.edu/map.html). By late 2020, it became apparent that the SARS-CoV-2 virus was acquiring mutations in the spike protein gene (S-gene) that impacted key biological and epidemiological properties of the virus, including viral transmissibility, infectivity, and immune escape (Ramesh et al., 2021; Tao et al., 2021b). Subsequently, there was rapid and sequential emergence of several variants of concern harboring clusters of S-gene mutations that defined new viral lineages. These included the Alpha (B.1.1.7), Beta (B.1.351), and Gamma (P.1) variants (Thye et al., 2021; Yi et al., 2021), followed by the Delta (B.1.617.2 and AY sublineages) variant; the Delta variant constituted over 99% of cases in the US toward the end of 2021, until the rapid emergence and spread of the new Omicron (BA.1) variant in December 2021 (https://covid.cdc.gov/covid-data-tracker).

At various times in 2020 and 2021, these SARS-CoV-2 variants have achieved the designation of variants of concern (VOC) as determined by the WHO and the United States Center for Disease Control and Prevention (CDC). A VOC is defined as a variant for which there is evidence of an increase in transmissibility, more severe disease (e.g., increased hospitalizations or deaths), significant reduction in neutralization by antibodies generated during previous infection or vaccination, reduced effectiveness of treatments or vaccines, or diagnostic detection failures (https://www.cdc.gov/coronavirus/2019-ncov/variants/variant-info.html). The WHO also classifies several additional strains as variants of interest (VOI), based on characteristics such as genetic changes that are predicted or known to affect virus characteristics (e.g., transmissibility, disease severity, immune escape) (https://www.who.int/en/activities/tracking-SARS-CoV-2-variants); the CDC has adopted the designation of variants being monitored (VBM) for such strains.

Whole genome sequencing (WGS), which has been employed by public health and other high-complexity laboratories, informs public health decisions by facilitating the tracking of SARS-CoV-2 transmission, outbreak detection, and contact tracking, and has been used to help trace the origin of the pandemic (Banerjee et al., 2021; Ferdinand et al., 2021; Park et al., 2021). With the sequential appearance of VOCs came the realization that, to mitigate the future impact of SARS-CoV-2 evolution, near “real time” identification of viral variants would be needed to rapidly determine the public health impact of emerging strains. Most laboratories performing SARS-CoV-2 real-time PCR testing, however, lack the ability to perform WGS. In addition, the cost and limited throughput of WGS places a limit on the number of samples being characterized during each wave of the pandemic. The availability of a rapid, accurate, and inexpensive real-time PCR-based approach for identifying sentinel mutations in the viral S-gene can enable the undertaking of much more comprehensive assessments of viral populations, followed by referral of select samples with atypical mutation profiles to central laboratories for complete genotypic analysis (Greninger et al., 2021).

In the present study, we describe the analytical performance characteristics of multiplexed assays for detection of the S-gene mutations E484K, N501Y, and S982A (PlexPrime^®^ SARS-CoV-2 Alpha/Beta/Gamma and Alpha/Beta/Gamma+, including Omicron), P681R (PlexPrime^®^ SARS-CoV-2 P681R Delta), and L452Q (PlexPrime^®^ SARS-CoV-2 L452Q Lambda). We further show these assays permit the rapid and accurate identification of SARS-CoV-2 viruses designated as VOC or VBM. Implementation of such testing could facilitate widespread, ongoing monitoring of SARS-CoV-2 evolution and early detection of variants requiring an urgent public health response and appropriate patient management.

## Methods

### Sample Collection

We obtained remnant RNA stored at -80°C, which had been extracted from deidentified clinical specimens previously tested for SARS-CoV-2 by quantitative reverse-transcription-PCR (qRT-PCR) at Quest Diagnostics and with variants previously assigned by WGS (Rosenthal et al., 2022). In accordance with ethical requirements and US Department of Health and Human Services guidelines for the use of deidentified specimen remnants in research studies, specimens were deidentified prior to the study and were limited to remnant specimens previously submitted for commercial testing.

### PlexPrime Methodology

The PlexPrime variant assays (SpeeDx Pty Ltd, Sydney, Australia) allow simultaneous detection of the SARS-CoV-2 pathogen *via* the RdRp gene and mutations associated with these variants (**Table 1**). The assays evaluated in this study included the initial version of the PlexPrime^®^ SARS-CoV-2 Alpha/Beta/Gamma assay as well as the expanded PlexPrime^®^ SARS-CoV-2 Alpha/Beta/Gamma+ that also includes the Omicron variant, PlexPrime^®^ SARS-CoV-2 P681R Delta, and PlexPrime^®^ SARS-CoV-2 L452Q Lambda. The Alpha/Beta/Gamma assay contains probes targeting the spike E484K, N501Y, and S982A mutations. The Alpha/Beta/Gamma+ assay targets the same mutations and includes an additional N501Y probe that accounts for mutations adjacent to spike position 501 in the Omicron variant. The assays all follow the same protocol, whereby respiratory samples or controls are extracted on the MagNA Pure 96 instrument (Roche Diagnostics, US). Briefly, 200 µL of sample was extracted and eluted in 50 µL with the DNA and Viral RNA Small Volume extraction kit using the Pathogen Universal 200 protocol (Roche Diagnostics). For the PlexPrime^®^ SARS-CoV-2 Alpha/Beta/Gamma, PlexPrime^®^ SARS-CoV-2 P681R Delta, and PlexPrime^®^ SARS-CoV-2 L452Q Lambda, a 10-µL aliquot of nucleic acid extract was added to a 15-µL reaction mix (12.5 µL Plex Mastermix, 1.25 µL of 20x SARS-CoV-2 variant oligo mix, 0.25 µL of 100x reverse transcriptase, 0.5 µL 50x RNase inhibitor, and 0.5 µL of nuclease-free water, for a final reaction volume of 25 μL) and tested in a 96-well plate. For the PlexPrime^®^ SARS-CoV-2 Alpha/Beta/Gamma+ with N501Y-Omicron assay, a second set of 0.5 µL of 20x SARS-CoV-2 variant oligo mix for N501Y-Omicron was added, instead of 0.5 µL of nuclease-free water. The PCR cycle was performed on an ABI 7500 FAST Dx Instrument (Thermofisher, US). Cycling consisted of 48°C for 10 min and 95°C for 2 min, followed by 10 cycles of 95°C for 5 s and 61°C for 30 s (−0.5°C per cycle) and 40 cycles of 95°C for 5 s and 52°C for 50 s. For the PlexPrime^®^ SARS-CoV-2 Alpha/Beta/Gamma assay and PlexPrime^®^ SARS-CoV-2 Alpha/Beta/Gamma+ assay, data acquisition was set for E484K in channel A (FAM), RdRp in channel B (JOE), S982A in channel C (Texas Red), and N501Y in channel D (Cy5). For the PlexPrime^®^ SARS-CoV-2 P681R Delta assay, data acquisition was set for RdRp in channel A (JOE) and P681R in channel B (Cy5). For the PlexPrime^®^ SARS-CoV-2 L452Q Lambda assay, data acquisition was set for RdRp in channel A (JOE) and L452Q in channel B (Texas Red). Analysis of the results was performed using the instrument software and an Excel spreadsheet to calculate ΔCqs (quantification cycles) between the RdRp channel and each mutation channel. The cutoff ranges were evaluated through analytical sensitivity studies using extracted clinical specimens that had undergone WGS and were set to specifically determine the mutations present, as follows:

**Table 1:** VBM and VOC spike gene mutations detected in SARS-CoV-2 PlexPrime^®^ assays^1^.

PlexPrime^®^ SARS-CoV-2	Alpha/Beta/Gamma/Alpha/Beta/Gamma+			P681R Delta	L452Q Lambda
Mutations	E484K	N501Y	S982A	P681R	L452Q
Alpha (B.1.1.7)		X	X		
Beta (B.1.351)	X	X			
Gamma (P.1)	X	X			
Delta (B.1.617.2, AY)				X	
Kappa (B.1.617.1)				X	
Lambda (C.37)					X
Omicron (BA.1, BA.2)		X			

^1^VBM, variant being monitored; VOC, variant of concern.

ΔCq E484K = E484K Ct – RdRp Ct; cutoff range: -2.0 to 5.0

ΔCq N501Y^1^ = N501Y Ct – RdRp Ct; cutoff range: -2.0 to 4.0

ΔCq N501Y^2^ = N501Y Ct – RdRp Ct; cutoff range: -2.5 to 4.0

ΔCq S982A = S982A Ct – RdRp Ct; cutoff range: -2.0 to 5.0

ΔCq P681R = P681R Ct – RdRp Ct; cutoff range: -2.0 to 5.5

ΔCq L452Q = L452Q Ct – RdRp Ct; cutoff range: -2.0 to 5.0

^1^ N501Y in the Alpha/Beta/Gamma assay.

^2^ N501Y in the Alpha/Beta/Gamma+ assay.

If the RdRp cycle threshold (Ct) value was > 19 for the E484K, N501Y, and S982A mutations in the Alpha/Beta/Gamma assay or >26 in the Alpha/Beta/Gamma+ assay, the result was inconclusive and the PlexPrime assay was repeated from sample extraction. Because we observed that the E484K probe could cross-react with the uncommon E484Q mutation, a positive result for this probe was considered as positive for E484K/Q. If the RdRp cycle threshold (Ct) value was > 26 for the P681R Delta or the L452Q Lambda mutations, the result was considered inconclusive and the PlexPrime assay was repeated from the extraction step.

### Analytical Sensitivity

Positive samples were extracted and tested with the Quest Diagnostic SARS-CoV-2 qualitative RT-PCR assay. An IFU for the assay can be found at the FDA website (https://www.fda.gov/medical-devices/coronavirus-disease-2019-covid-19-emergency-use-authorizations-medical-devices/in-vitro-diagnostics-euas-molecular-diagnostic-tests-sars-cov-2). Primer and probes for the Quest SARS-CoV-2 assay are based on the CDC primer and probe sets 2019-nCoV_N1 and 2019_nCoV_N3 (https://www.cdc.gov/coronavirus/2019-ncov/downloads/rt-pcr-panel-primer-probes.pdf). Samples were quantitated using a standard curve generated by serially diluting the SARS-CoV-2 viral transcript containing the N gene in stabilizing buffer consisting of poly-A RNA in PBS. Sample dilutions were made in their respective negative sample matrix, including viral transport media (UTM, Becton-Dickenson) and phosphate-buffered saline (PBS). For each targeted mutation, 20 replicates were tested at 6 different concentrations. The limit of detection was defined as the lowest viral load (copies/mL) that resulted in 95% detection of the mutation in all replicates assayed.

### Precision

Intra-assay precision was assessed for each lineage in triplicate for high, medium, and low viral load standards diluted in PBS or UTM and was defined as the percent coefficient of variation (CV) for the triplicate Ct values (**Supplementary Table S1**). Similarly, inter-assay precision was assessed by testing the inter-assay precision plate in two independent runs on different days.

### Analytical Specificity

To verify that the PlexPrime SARS-CoV-2 variant assays did not detect viral nucleic acid from other related respiratory pathogens, the PlexPrime assays were tested on the ZeptoMetrix NATtrol™ Respiratory Verification Panel 2.1 (#NATRVP2.1-BIO), a panel comprised of individual inactivated respiratory related pathogens (purified, intact virus particles and bacterial cells) manufactured specifically for use as positive controls in nucleic acid tests (**Supplementary Table S2**).

### Accuracy

To assess accuracy of RT-PCR genotyping, variant results were compared to those from WGS (Rosenthal et al., 2022). Proportions were compared with the Fisher Exact test.

## Results

### Assay Sensitivity

Dilution series of SARS-CoV-2 Alpha (B.1.1.7) in two different collection media, UTM and PBS, were prepared to assess the sensitivity for the detection of spike gene mutations N501Y and S982A using the Alpha/Beta/Gamma+ assay. SARS-CoV-2 gamma (P.1) was selected for sensitivity studies of mutations N501Y and E484K in the Alpha/Beta/Gamma+ assay. Sensitivity for mutations was assessed using SARS-CoV-2 Delta (B.1.617.2) for the P681R mutation and SARS-CoV-2 lambda (C.37) for L454Q. Sensitivity for N501Y in the Alpha/Beta/Gamma+ assay was assessed using SARS-CoV-2 Omicron (BA.1). The limits of detection for the respective targets and media types ranged from 500 copies/mL for the N501Y and S982A mutation in lineage B.1.1.7 in UTM or PBS, to a maximum of 10,000 copies/mL for the E484K mutation in lineage P.1 diluted in UTM (**Table 2**).

**Table 2:** Assay sensitivity for the detection of targeted spike gene mutations^1^.

Lineage	Target	Sensitivity (copies/mL)	
		PBS	UTM
Wuhan strain^2^ (Wild type)	RdRp	1000	2500
Alpha (B.1.1.7)^2^	RdRp	500	500
	N501Y	1000	1000
	S982A	1000	500
Gamma (P.1)^3^	RdRp	500	500
	N501Y	2000	1000
	E484K	4000	10000
Delta (B.1.617.2)	RdRp	2500	1000
	P681R	2500	2500
Lambda (C.37)	RdRp	500	1000
	L452Q	2500	1000
Omicron (BA.1)^3^	RdRp	1000	1000
	N501Y	1000	1000

^1^RdRp, RNA dependent RNA polymerase target common to all SARS-CoV-2 lineages. ΔCq = target Ct – RdRp Ct, NA, not applicable.

^2^Seracare product number 0505-0241.

^3^Alpha/Beta/Gamma+ kit is supplemented with an Omicron-specific N501Y probe that is distinct from the Alpha/Beta/Gamma N501Y probe in the original Alpha/Beta/Gamma kit.

### Reproducibility

The maximum percentage of CVs of the replicate Ct values for the RdRp gene and for 5 spike gene variants assessed for intra-assay reproducibility was <4% for all mutations and media types and viral load levels. Likewise, the maximum percentage of CVs assessed for inter-assay reproducibility in 3 runs was <7% for all mutations and media types and viral load levels (**Supplementary Tables S3, S4**).

### Analytical Specificity

To verify that the variant assays do not detect nucleic acids from other common coronaviruses and respiratory pathogens, we tested a panel that included material from 32 non-SARS-CoV-2 pathogens, including the 4 common human coronaviruses 229E, HKU-1, NL63, and OC43 (**Supplementary Table S2**). No cross-reactivity was seen for any of the non-SARS-CoV-2 pathogens. The SARS-CoV-2 strain WA1/2020 yielded a positive RdRp result for all PlexPrime tests, as expected.

### Accuracy (Blind Panel)

To assess accuracy of the Alpha/Beta/Gamma assay, we constructed blind panels of remnant extracted nucleic acid from SARS-CoV-2 positive patient samples that were typed by WGS. These panels encompassed 30 different lineages, including multiple lineages harboring the E484K, S982A, and N501Y mutations (**Supplementary Table S5**). For the Alpha/Beta/Gamma Variant Assay, 2 specimens with lineage B.1.526 out of 398 positive specimens tested gave discordant results, with positive detection for N501Y, E484K, and S982A. An additional 2 specimens (B.1.1.7 and P.1 lineages) had inconclusive results (RdRp Ct > 19), and 4 specimens had invalid results with undetermined RdRp Cts (**Table 3**). Upon repeat extraction from the original specimen tubes and retesting, one B.1.526-discordant specimen was resolved as negative for E484K, N501Y, and S982A, and the inconclusive B.1.1.7 specimen was confirmed to have the expected mutations. Four specimens were inconclusive and 2 gave invalid results. There were no further discordant results.

**Table 3:** Accuracy of the SARS-CoV-2 PlexPrime assays relative to WGS^1^.

	SARS-CoV-2 Status	Total	Concordant	Discordant	Inconclusive^2^	Invalid	Agreement(%)
Alpha/Beta/ Gamma	Positive	398	392	0	4	2	100
	Negative^6^	39	39	0	0	0	100
Delta^3^	Positive	664	656	4	4	0	99.4
	Negative^6^	17	17	0	0	0	100
Lambda^4^	Positive	578	568	1	9	0	99.8
	Negative^6^	16	16	0	0	0	100
Alpha/Beta/ Gamma+^5^	Positive	57	55	1	1	0	98.2
	Negative^6^	5	5	0	0	0	100
Totals		1774	1748	6	18	2	99.7

^1^Accuracy was defined as the presence of the Spike E484K (Beta, Gamma), N501Y (Alpha, Beta, Gamma, Omicron), and S982A (Alpha), P681R (Delta), and L452Q (Lambda) mutations. Agreement (%) = (#concordant)/(#total - #invalid - #inconclusive).

^2^RdRp Ct value > 19 [Alpha/Beta/Gamma assay or >26 (other assays)].

^3^394/664 positives were in Delta lineages B.1.617.2 and AY sublineages.

^4^Only a single Lambda specimen was available for the accuracy panel.

^5^Positives included a blind panel of 16 Omicron and 16 Delta clinical specimens typed by WGS as well as 5 high (25,000 copies/mL), 4 medium (10,000 copies/mL), and 5 low (2500 copies/mL) viral load diluted Omicron specimens.

^6^Negatives included SARS-CoV-2 negative patient specimens and no-template controls.

We further constructed an accuracy panel to assess the performance of the Delta and Lambda PlexPrime assays. A total of 664 SARS-CoV-2 positive specimens collected in June, July, and August 2021 were randomly selected. These included 394 classified as Delta by WGS (**Supplementary Table S6**). Of the total, 656 variant assay results were concordant with WGS for an overall 99.4% agreement following repeat testing (**Table 3**). From the initial 10 discordant specimens, which consisted of 9 Delta lineage specimens for which P681R was not detected and one B.1.637 lineage specimen that was positive for P681R, 7 of 10 discordant specimens were available for repeat testing. Of these, 5 were resolved to a detected result for P681R; one sample was inconclusive, and one remained undetected. As SARS-CoV-2 Lambda (C.37) is uncommon in the United States, only a single Lambda specimen was available for testing. The L452Q spike mutation was successfully detected in the Lambda variant assay for this specimen and was not detected in 577 non-Lambda lineages (**Table 3**). To further assess the Lambda variant assay, we constructed a dilution series of a Lambda specimen in UTM or PBS media at concentrations of 5000 to 60,000 copies/mL, as well as a non-Lambda (“WT”) specimen at 50,000 copies/mL. A blind panel was constructed consisting of 10 replicates at each concentration in UTM and PBS. The L452Q spike mutation was detected in all Lambda replicates and in none of the WT replicates, demonstrating that the L452Q mutation can be reliably detected in Lambda specimens.

An additional accuracy panel was tested to assess the performance of the Alpha/Beta/Gamma+ assay for detecting the Omicron variant. The blind panel included 16 Omicron (lineage BA.1), 16 Delta clinical specimens, 3 Alpha (lineage B.1.1.7), 4 Gamma (lineage P.1), and 4 Wuhan WT typed by WGS, as well as 5 high (25,000 copies/mL), 4 medium (10,000 copies/mL), and 5 low (2500 copies/mL) viral load diluted Omicron specimens (**Supplementary Table S7**). Overall positive percentage agreement was 98.2% (**Table 3**). There was one inconclusive result for a low-positive Omicron sample with an RdRp Ct > 26, and one discordant result for the E484K/Q mutation in a P.1 specimen (ΔCq E484K = 5.18; cutoff: 5.0). In addition to the blind panel, 4 clinical specimens determined by WGS to belong to Omicron lineage BA.2 were assessed with the Alpha/Beta/Gamma+ assay and all 4 were detected with low N501Y Delta Ct values between 0.85 and 2.17.

**Figure 1:**
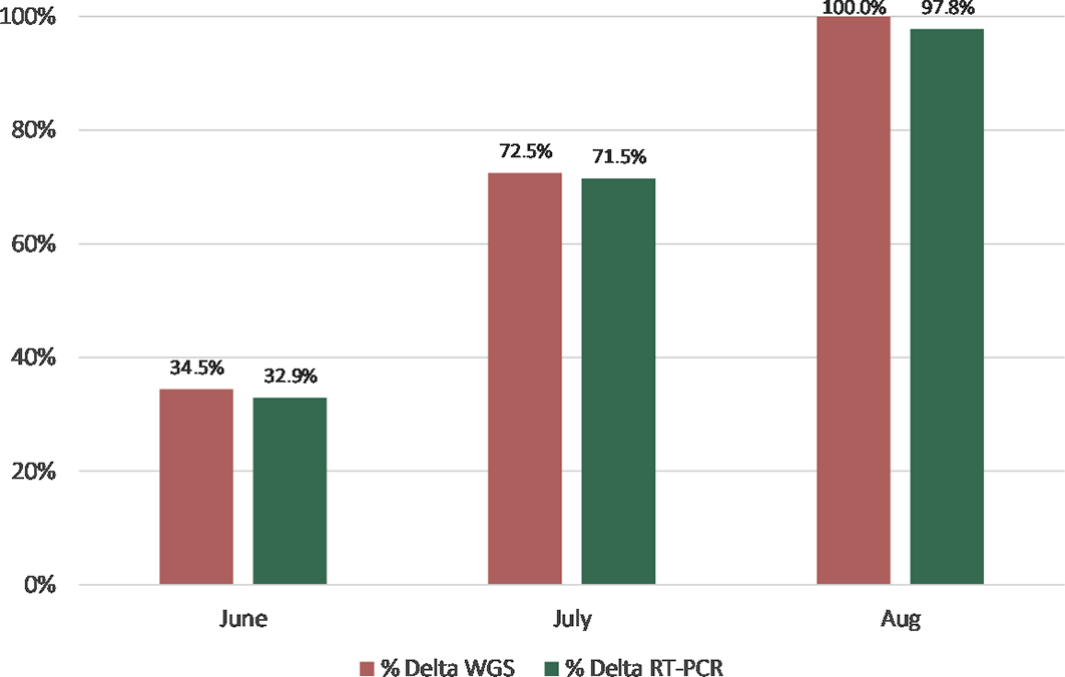
Prevalence of the Delta lineage (June-August 2021) by WGS vs. RT-PCR genotyping (N= 664). The differences in the Delta lineage proportions between WGS and RT-PCR were not statistically significant (Fisher Exact test).

For the WGS data, the prevalence of the Delta lineage increased from 34.5% to 100% over the 3-month period in which these samples were collected (**Figure 1**). The prevalence of the lineage-defining P681R mutation as assessed in the Delta variant assay also increased over the same period and closely tracked the Delta lineage prevalence as determined by WGS (**Figure 1**). The prevalence differences for the Delta lineage between WGS and RT-PCR (June: 0.015 [-0.057 – 0.87]; July: 0.010 [-0.078 – 0.098]; August: 0.022 [-0.006 - 0.063]) were not statistically significant (Fisher Exact test).

## Discussion

The surveillance of SARS CoV-2 variants during the pandemic has been primarily driven by WGS. While WGS remains the gold standard for genotyping, its availability is limited to laboratories with next generation sequencing and bioinformatics capabilities. Rapid variant typing by RT-PCR provides a high throughput method to enhance surveillance efforts as well as provide information that can guide infection control and treatment decisions in a timely manner (https://www.covid19treatmentguidelines.nih.gov/management/clinical-management/nonhospitalized-adults–therapeutic-management/). In this study, we have described and characterized the performance of a genotypic assay that can facilitate the rapid and accurate identification of VOC and VBM designated SARS-CoV-2 viruses.

Variant screening by RT-PCR has been described in the literature (Adamoski et al., 2021; Bedotto et al., 2021; Hamill et al., 2021; Lind et al., 2021; Migueres et al., 2021; Park et al., 2021; Perchetti et al., 2021; Vega-Magaña et al., 2021; Wang et al., 2021; Garson et al., 2022; Peterson et al., 2022). Wang et al. applied a multiplex allele-specific reverse transcriptase PCR (RT-qPCR) to detect three spike protein mutations (L452R, E484K, N501Y) (Wang et al., 2021). Assay specificity was validated with WGS and identified the rapid emergence of the L452R within the San Francisco Bay area population. While RT-PCR can provide high-throughput genotyping in laboratories already performing SARS-CoV-2 molecular testing, it has not been widely implemented in the United States, and no commercial assays have yet received emergency use authorization (EUA) from the US Food and Drug Administration. The rapid emergence of the Omicron variant and the limited use of monoclonal antibodies to treat patients harboring this variant while the Delta variant is still circulating highlight the role for rapid genotyping in clinical care. An Infectious Disease Society of America (IDSA) and American Society for Microbiology (ASM) consensus review provided an overview of the clinical and infection prevention applications of SARS-CoV-2 genotyping, including suggested examples of reporting variant typing results (Greninger et al., 2021).

In this study, the overall agreement between variant typing and WGS was > 99%. The RdRp target served as an internal control for known positives as well as an indicator of target load to assure mutation detection. Among the 1774 accuracy panel members tested, only 18 specimens (1.01%, **Table 3**) were deemed inconclusive for mutation analysis based on the RdRp Cq and would be repeated in clinical samples. We postulate that some of the discordant results may be attributable to viral RNA degradation or cross-sample contamination.

From June to August 2021, the Delta variant emerged in the US displacing the Alpha variant as the dominant circulating strain. The sampling of specimens with the Delta variant typing assay correlated with WGS (**Figure 1**) and could have provided more real-time surveillance by reflexing positive SARS-CoV-2 samples to the typing assay. At the time of this writing, the Omicron variant was spreading globally; thus, the Delta typing assay could still be utilized to look for shifts in the percentage of Delta cases as well as to guide appropriate treatment therapies (https://www.covid19treatmentguidelines.nih.gov/management/clinical-management/nonhospitalized-adults–therapeutic-management/).

There are limitations to utilizing an RT-PCR typing assay. Given that such assays focus on a select group of mutations, the results are preliminary and may require additional genotyping for confirmation or to provide a richer epidemiological picture. However, if the primary circulating strain is dominant in a population (e.g., the Delta or the Omicron variant circulating at >99%), the need for confirmation by sequencing may be minimal. Rapid SARS-CoV-2 viral evolution and the imperative to address diagnostic needs are challenging. As new variants emerge, there is a need to update the assay design for typing (Greninger et al., 2021). Despite these limitations, a rapid RT-PCR genotyping assay would allow a wider net to be cast for surveillance and serve as an early warning signal when there is a shift in the circulating variants, which may cause greater disease burden or medical intervention failures. Additionally, a genotyping assay may have utility to distinguish between persistent infection with the same viral strain vs. reinfection due to a new strain in an individual with recurrent positive molecular test results for SARS-CoV-2. Finally, a genotyping assay could be designed to detect select variants in the spike (S), RdRp polymerase, or the nsp5 protease coding regions associated with reduced efficacy to therapeutics including spike-targeting monoclonal antibodies (Kreuzberger et al., 2021), polymerase inhibitors such as remdesivir (Tao et al., 2021a) and molnupiravir (Pourkarim et al., 2022), and protease inhibitors such as nirmatrelvir (Vangeel et al., 2022). An RT-PCR genotyping assay may be performed in a high-throughput manner and at a lower cost than WGS. CPT coding for SARS-CoV-2 genotyping to ensure that US laboratories are reimbursed for the testing being performed became available as of February 21, 2022, with the release of CPT code 87913, covering SARS-CoV-2 mutation identification in targeted region(s) (https://www.ama-assn.org/system/files/coronavirus-long-descriptors.pdf). At present, the clinical use case for a rapid genotyping assay is to guide antiviral therapy (Greninger et al., 2021 and https://www.covid19treatmentguidelines.nih.gov/management/clinical-management/nonhospitalized-adults–therapeutic-management/). With expanding therapies available for SARS-CoV-2, the utility of diagnostics to guide therapy will likely expand.

In summary, this study demonstrates the performance and utility of RT-PCR genotyping assays. Such assays could be more widely available to laboratories performing SARS-CoV-2 molecular testing if a commercially available solution were available with regulatory approval.

## Data Availability Statement

The raw data supporting the conclusions of this article will be made available by the authors, without undue reservation.

## Author Contributions

GB performed all experiments and participated in data analysis, study design, and manuscript review and editing. RK participated in data analysis, data review, manuscript preparation, and editing. EM participated in the study conception and design, data review, manuscript preparation, and editing. All authors contributed to the article and approved the submitted version.

## Funding

This study received funding from Quest Diagnostics. The funder was not involved in the study design, collection, analysis, interpretation of data, the writing of this article, or the decision to submit it for publication. All authors declare no other competing interests.

## Conflict of Interest

The authors are employees of Quest Diagnostics, a commercial reference laboratory that performs commercial SARS-CoV-2 testing and own shares of Quest Diagnostics stock.

## Publisher’s Note

All claims expressed in this article are solely those of the authors and do not necessarily represent those of their affiliated organizations, or those of the publisher, the editors and the reviewers. Any product that may be evaluated in this article, or claim that may be made by its manufacturer, is not guaranteed or endorsed by the publisher.
